# Bioaugmented methanol production using ammonia oxidizing bacteria in a continuous flow process

**DOI:** 10.1016/j.biortech.2019.01.092

**Published:** 2019-05

**Authors:** Yu-Chen Su, Sandeep Sathyamoorthy, Kartik Chandran

**Affiliations:** Columbia University, Department of Earth and Environmental Engineering, 500 West 120th Street, Room 1045 Mudd Hall, New York, NY 10027, United States

**Keywords:** Ammonia oxidizing bacteria, Methanol, Resource recovery

## Abstract

Organic compounds such as methanol are widely used for enhancing denitrification at wastewater treatment plants (WWTPs) to meet effluent water quality permits. On the other hand, methane, which is the main feedstock for industrial methanol production, is also generated during anaerobic digestion in WWTPs, but is often flared to mitigate its greenhouse impacts. The overarching goal herein was to develop a novel continuous process for methanol production from methane using nitrifying activated sludge. The maximum AOB specific methanol production rate using hydroxylamine as electron donor was 1.61 ± 0.15 and 1.27 ± 0.15 mg-COD-CH3OH/(mg-COD-AOB*d), for hydraulic retention times (HRTs) of 7.5 h and 2 h, respectively. The corresponding production rate using ammonia as electron donor was 0.31 ± 0.08 mg-COD-CH3OH/(mg-COD-AOB*d) at a HRT of 2 h. These results show that nitrifier-mediated methanol production in a continuous-flow system can enhance the efficiency of WWTPs through internal production of biomethanol for denitrification, while simultaneously minimizing wasteful biogas flaring.

## Highlights

• Bioaugmented process for carbon production at water resource recovery facilities.
• Single-step bioconversion of methane to methanol.
• Reduced chemical cost and improved sustainability of biological nitrogen removal.
• Ready integration into existing water resource recovery facility configurations.

## Graphical abstract

**Figure:**
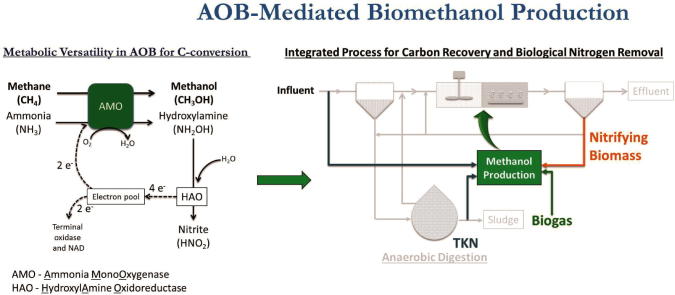

## 1 Introduction

There is a global effort to expand the traditional role of wastewater treatment facilities to integrate recovery of resources. These efforts have in the past primarily relied on beneficial reuse of water and biosolids. However, recent research has focused on the production of resources including nutrients for fertilizer production (El Diwani et al., 2007, Desmidt et al., 2015), biofuels (Kargbo, 2010), bioplastics (Rodgers and Wu, 2010), and commodity chemicals including methanol (Taher and Chandran, 2013). Methanol is the most widely used exogenous carbon source in water resource recovery facilities (WRRFs) for denitrification to achieve low total nitrogen levels (Cherchi et al., 2009). However, the cost of methanol for denitrification is dependent on competing demand from industries such as utility chemicals manufacturing. Furthermore, in some cases, safety concerns related to methanol storage and handling facilities might preclude the purchase and transport of commercial methanol to WRRFs, highlighted by the ban of methanol use for denitrification in New York City in the late 2000s. Therefore, biogenic production of methanol and *in-situ* utilization within a WRRF presents a particularly valuable opportunity to reduce the dependence on external carbon sources and enhance overall WRRF sustainability.

**Fig. 1:**
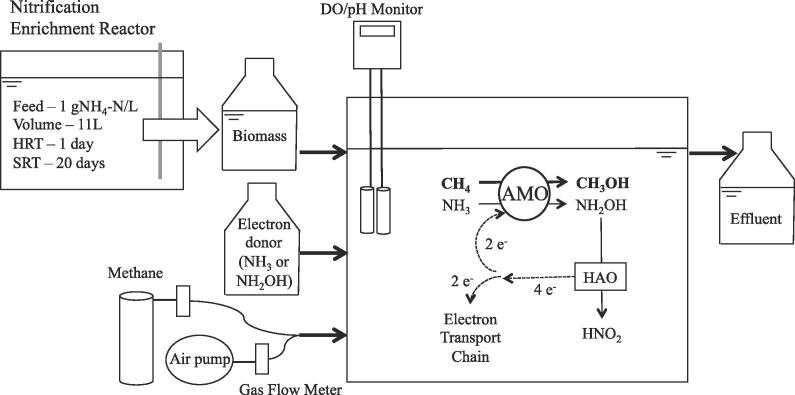
Experimental setup of the CSTR for methanol production. Shown in the reactor, for reference purposes, is the conversion of ammonia and methane as well as the electron flow within AOB.

Conversion of methane to methanol by ammonia oxidizing bacteria (AOB) is facilitated by the ammonia monooxygenase (AMO) enzyme, which shares similar characteristics with particulate methane monooxygenase (pMMO) in methane oxidizing bacteria (MOB) (Holmes et al., 1995). The oxidation of ammonia (NH_3_) (Eq. (1)) or methane (Eq. (3)), a competing substrate for ammonia (Hyman and Wood, 1983), requires two electrons which are generated through the oxidation of hydroxylamine (NH_2_OH) to nitrite (NO_2_^−^) (Eq. (2)) (Prosser, 1989) (Fig. 1).

(1) $${NH}_{3}+O_{2}+{2H}^{+}+{2e}^{-}\to {NH}_{2}OH+H_{2}O$$

(2) $${NH}_{2}OH+H_{2}O\to {NO}_{2}^{-}+{5H}^{+}+{4e}^{-}$$

(3) $${CH}_{4}+O_{2}+{2H}^{+}+{2e}^{-}\to {CH}_{3}OH+H_{2}O$$

For MOB, methanol, which is the intermediate for energy metabolism and carbon assimilation pathways, is further oxidized by methanol dehydrogenase (MDH) (Hanson and Hanson, 1996). In order to achieve selective oxidation of methane to methanol in MOB, addition of MDH inhibitors has been reported (Ge et al., 2014). On the other hand, there are no known enzymes for methanol metabolism present in the genomes of common AOB (Chain et al., 2003, Stein et al., 2007) thereby rendering them an attractive alternative platform for biomethanol production. Biogenic conversion of methane to methanol has been previously demonstrated at the laboratory scale, using mixed and axenic cultures of AOB (Hyman and Wood, 1983, Wang et al., 2010, Taher and Chandran, 2013) fed with either NH_3_ or NH_2_OH. A recent study has demonstrated especially high rates and yield of biomethanol production using NH_2_OH as the electron donor (Taher and Chandran, 2013). The process benefit of using NH_2_OH as the electron donor rather than NH_3_, is the lack of competition for reducing equivalents with methane. However, hydroxylamine needs to be purchased while ammonia is readily available in WRRFs either in the mainstream sewage itself or in sidestreams such as anaerobic digestion dewatering post-centrifugation (centrate) or post-filtration (filtrate) streams. Although integration of bioconversion of methane to methanol in WRRFs holds promise, important questions remain as to the efficacy and viability of such a process using continuous cultures to generate significant methanol yields. Additionally, biogenic methanol production has not been demonstrated using continuous flow bioreactors.

The overall objective of this research was to evaluate biogenic production of methanol using a mixed nitrifying enrichment culture in a continuous stirred tank reactor (CSTR) and compare the methanol production performance using either ammonia or hydroxylamine as the electron donor.

## 2 Material and methods

### 2.1 Nitrification enrichment culture

A nitrifying enrichment culture was developed in a 11.2 L parent reactor to provide active nitrifying bacteria for the methanol production studies. The parent reactor feed contained: 1000 mgNH_3_-N L^−1^, 3.3 mg L^−1^ FeSO_4_·7H_2_O, 3.3 mg L^−1^ MnSO_4_·7H_2_O, 0.7 mg L^−1^ (NH_4_)_6_Mo_7_O_24_·4H_2_O, 0.8 mg L^−1^ CuCl_2_·2H_2_O, 3.0 mg L^−1^ ZnSO_4_·7H_2_O, 0.6 mg L^−1^ NiSO_4_·6H_2_O, 0.3 g L^−1^ MgSO_4_·7H_2_O, 0.2 g L^−1^ KH_2_PO_4_, 0.5 g L^−1^ K_2_HPO_4_, and 0.7 g L^−1^ NaHCO_3_. The parent reactor was operated at room temperature (23 ± 1 °C) with a hydraulic retention time (HRT) and a target solids retention time (SRT) of 1 day and 20 days, respectively. The parent reactor dissolved oxygen (DO) concentration was measured (YSI, Yellow Spring, OH) and maintained at 3–4 mg L^−1^ using filtered lab air (0.2 μm, Whatman). Parent reactor pH was continuously monitored and maintained at 7.50 ± 0.05 using 1 M NaHCO_3_ (Etatron dosing system, Clearwater, FL). Parent reactor performance was regularly monitored by tracking influent NH_3_, reactor COD and effluent NH_3_, nitrite and nitrate concentrations and full nitrification was maintained.

### 2.2 Methanol production experiments

Methanol production experiments were designed to test different electron donors (NH_3_ or NH_2_OH) and hydraulic retention times (7.5 h or 2 h) to evaluate the rates and yields of methanol production. The experiments were conducted in a 1.5-liter test CSTR (Fig. 1) at ambient lab temperature (23 ± 1 °C). Four liters of mixed liquor from the parent nitrification enrichment reactor were collected and prepared for use in the methanol production CSTR by twice centrifuging (4000×*g*, 5  min) and washing in a nitrogen-free medium. The washed biomass was re-suspended in the nitrogen-free medium to achieve a target COD of 1000 mg L^−1^. A unique CSTR design was employed wherein nitrifying biomass was bioaugmented into the CSTR (Fig. 1), to mimic integrated mainstream-sidestream nitrogen treatment practiced currently in increasingly more WRRFs while simultaneously mitigating inhibition of AOB by the methanol produced. The target HRT of the biomethanol production reactor was achieved by controlling the biomass feed rate. In all experiments, the nitrogenous electron donor was prepared at a concentration of 589 mg-N L^−1^, which was based on the typical ammonia-N concentration range (500–800 mg-N/L) of in anaerobic digestion centrate (NYSERDA, 2007). Influent nitrogen was supplied to the CSTR using a syringe pump (Kent Scientific, Torrington, CT) at a rate of 11.4 ml h^−1^, resulting in a mass rate of 6.7 mg-N h^−1^. Air was provided using an air blower (Tetra, Blacksburg, VA) and 99.99% methane gas (PurityPlus 4.0, TechAir) was also provided. Gas flow rates were controlled using rotameters (Cole Parmer, Vernon Hills, IL). The air and methane flow rates were maintained at 0.1 L min^−1^ each in all experiments. All experiments were carried out under non-limiting DO conditions (above 1 mg O_2_ L^−1^). CSTR pH was continuously monitored (Jenco, San Diego, CA) and manually maintained at 7.50 ± 0.05 with 1 M NaHCO_3_. The HRT of 7.5 h was close to that of a previous study (Taher and Chandran, 2013) while the 2 h HRT was selected to reflect a practicable industrial scale system, for which both NH_3_ and NH_2_OH were evaluated as electron donors.

### 2.3 Analytical methods

Methanol concentration was determined using gas chromatography equipped with flame ionization detection (GC-FID, SRI Instruments, Torrance, CA). The GC was fitted with a 60 m × 0.53 mm ID × 5 μm df capillary MXT-1 column (Restek, Bellefonte, PA). The carrier gas was helium at a constant flow rate of 20 ml min^−1^ and the GC oven temperature was maintained at 120 °C. One microliter sample was injected manually through the on-column injection port. Ammonia nitrogen concentration was measured using gas-sensing combination Ion Selective Electrode (Thermo Fisher, Waltham, MA) for the 7.5 h HRT experiments, and a colorimetric assay (Method 10031, Hach, Loveland, CO) for the 2 h HRT experiments. Hydroxylamine was measured as described previously (Frear and Burrell, 1955). For the 7.5 h HRT experiments, nitrite and nitrate concentrations were measured using a colorimetric assay (APHA et al., 1999) (reagents purchased from Ricca chemical, Arlington, TX) and an Ion Selective Electrode (Thermo Fisher, Waltham, MA), respectively. For the 2 h HRT experiments, the concentrations of nitrite and nitrate were quantified using ion chromatography coupled with a conductivity detector (Dionex ICS 2100). Separation was achieved using a Dionex Ionpac AS-18 (2 × 250 mm) with a Dionex AG-18 guard column (2 × 50 mm) and isocratic separation at 0.25 ml min^−1^ using a 25 mM KOH eluent. COD was measured using a digestion method (Method 8000, Hach, Loveland, CO).

### 2.4 DNA extraction and quantification

Biomass DNA was extracted with a DNeasy mini kit on a Qiacube robotic workstation (Qiagen, Valencia, CA) following the manufacturer’s protocol. DNA concentration was subsequently measured using UV absorbance (NanoDrop Lite, Thermo Scientific, Waltham, MA). Extracted DNA was stored at −80 °C prior to further use. Quantitative real time polymerase chain reaction (qPCR) was used to quantify the gene copy concentrations of total eubacteria 16S rRNA gene (Ferris et al., 1996), AOB ammonia monooxygenase gene subunit A (Rotthauwe et al., 1997), *Nitrospira* spp. (Kindaichi et al., 2006) and *Nitrobacter* spp. (Graham et al., 2007) 16S rRNA gene. From qPCR results, the percentage of AOB, NOB and other heterotrophs in the biomass was determined. AOB concentrations were approximated by multiplying the active fraction of total COD and AOB percentage.

### 2.5 Methane to methanol conversion ratio

CH_4_ was supplied at a rate of 0.1 L/min, with an equimolar air:CH_4_ ratio. Using a molar gas volume of 22.414 L/mol, this corresponds to a CH_4_ supply rate of 133.84 mmol/h. In the absence of any biological conversion, the saturation CH_4_ concentration in the reactor fed with the equimolar air-CH_4_ gas mixture would be 0.71 mmol/L (for a 1:1 air:CH_4_ mixture). The gas-liquid mass-transfer coefficient (k_L_a) for CH_4_ (0.104 min^−1^) was experimentally determined using the same reactor setup. The following gas-liquid transfer rates for methane can be calculated using the expression:

$$Q_{CH4}=K_{L}a\times \left(C^{*}-C\right)\times V_{reactor}$$

where Q_CH4_ is the CH_4_ mass transfer rate (mmol/min), C* is the saturation concentration of CH_4_ (mmol/L), C is the CH_4_ concentration in the reactor (mmol/L), and V_reactor_ is the reactor volume (1.5 L).

CH_3_OH output rate was calculated using the expression:

$$Q_{CH3OH}=C_{CH3OH}\times Reactor\;effluent\;flow\;rate$$

where Q_CH3OH_ is the CH_3_OH output rate (mmol/min), C_CH3OH_ is the CH_3_OH concentration in the reactor after 6 h operation (mmol/L) when the methanol concentrations stabilized, and the reactor effluent flow rate (equal to the biomass feed rate) was 0.2 L/h or 0.75 L/h corresponding to the test HRTs of 7.5 h and 2 h, respectively. Hence, CH_4_ to CH_3_OH conversion ratio can be calculated as

$$\%\;{CH}_{4}\;supplied\;converted\;to\;{CH}_{3}OH=\frac{{CH}_{3}OH\;output\;rate}{{CH}_{4}\;supply\;rate}*100$$

or

$$\%\;{CH}_{4}\;transferred\;converted\;to\;{CH}_{3}OH=\frac{{CH}_{3}OH\;output\;rate}{{CH}_{4}\;mass\;transfer\;rate}*100$$

## 3 Results and discussion

### 3.1 Continuous methanol production using hydroxylamine as electron donor

Results from a previous study have shown that a higher biomethanol production rate and yield can be achieved when biomass was routinely replaced with fresh biomass every 2 h (Taher and Chandran, 2013). In that previous study, for one of the experimental conditions (termed ‘High-Rate’ therein), the biomass was separated from the spent reaction medium by filtration every two hours. Fresh biomass was collected from parent nitrifying reactor, washed with nitrogen-free medium, pelleted and re-suspended in new reaction medium. After biomass replacement, hydroxylamine and methane was fed into the system which resulted in increased methanol production relative to pre-replenishment. Therefore, for this study, the expectation was that methanol production rates and yields can be improved using a continuous biomass flow system in which methanol exposure time can be controlled. Accordingly, the continuous flow process design incorporated bioaugmentation of fresh nitrifying biomass into the biomethanol production reactor in a manner similar to how a mainstream nitrification process might be connected to sidestream nitrification process in a WRRF.

**Fig. 2:**
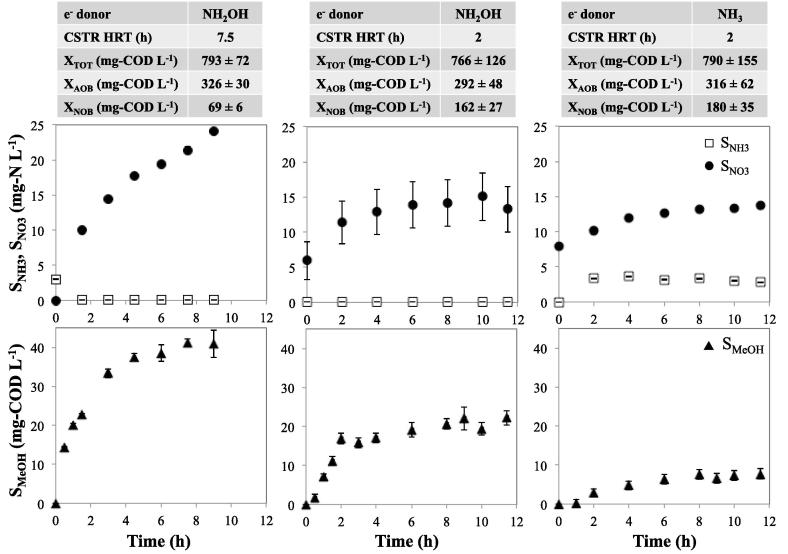
Measured concentrations of nitrogen species (top panels) and methanol (bottom panels) for 2 h and 7.5 h HRT CSTR experiments with hydroxylamine (left and middle panels) and ammonia (right panels) as the electron donor. Included above the figures are the AOB and NOB biomass concentrations from each experiment.

**Fig. 3:**
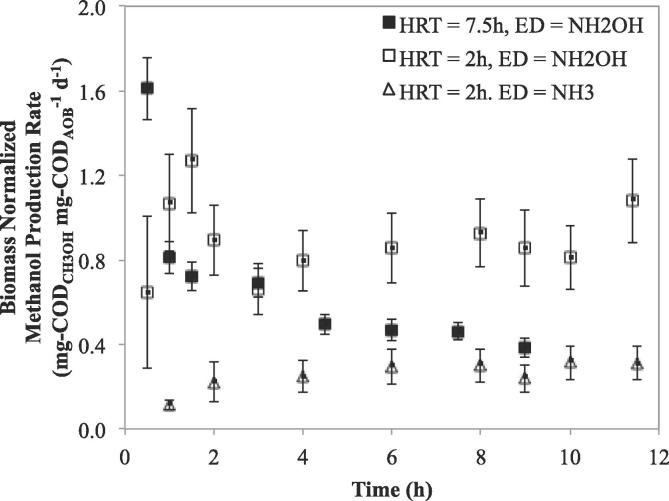
Biomass normalized methanol production rates for 2 h and 7.5 h HRT CSTR experiments with hydroxylamine and ammonia as the electron donor (ED).

The maximum CH_3_OH concentration achieved using NH_2_OH as the electron donor at a CSTR HRT of 7.5 h, was 41.0 ± 3.4 mg-COD_CH3OH_ L^−1^ (Fig. 2, bottom left panel). Operation of the CSTR at 2 h HRT resulted in a maximum CH_3_OH concentration of 21 ± 4.6 mg-COD_CH3OH_ L^−1^ (Fig. 2, bottom middle panel). Notwithstanding the different maximum methanol concentrations, the peak biomass specific CH_3_OH production rates (r_CH3OH−NH2OH_) were independent of CSTR HRT and in the range of 1.2–1.6 mg-COD_CH3OH_ mg-COD_AOB_^−1^ d^−1^ (Fig. 3). The steady state r_CH3OH−NH2OH_ with the CSTR operated with a 2 h HRT (0.91 ± 0.11 mg-COD_CH3OH_ mg-COD_AOB_^−1^ d^−1^, Table 1) was higher than the r_CH3OH−NH2OH_ with 7.5 h HRT (0.44 ± 0.05 mg-COD_CH3OH_ mg-COD_AOB_^−1^ d^−1^, Table 1). The lower r_CH3OH−NH2OH_ at the higher HRT is likely due to AMO inhibition resulting from longer exposure to methane or methanol in the CSTR, both of which are known AMO inhibitors (Jonsson et al., 2001, Taher and Chandran, 2013). Conversely, the higher rate of methanol production on hydroxylamine, r_CH3OH−NH2OH_ achieved with a lower HRT indicate that AMO inhibition can be effectively alleviated through appropriate process engineering measures such as HRT control.

**Table 1:** Summary of biogenic methanol production using mixed culture nitrifying biomass.

Electron Donor	HRT (hour)	Max S_MeOH_*1 mg-COD_CH3OH_ L^−1^	Max Biomass Normalized MeOH Production Rate mg-COD_CH3OH_ mg-COD_AOB_^−1^ d^−1^	Steady-State Biomass Normalized MeOH Prod. Rate*2 mg-COD_CH3OH_ mg-COD_AOB_^−1^ d^−1^	% methane supplied converted to methanol	% methane transferred converted to methanol
NH_2_OH	7.5	41.0 ± 3.4	1.61 ± 0.15	0.44 ± 0.05	0.13	2.6
NH_2_OH	2	21.0 ± 4.6	1.27 ± 0.15	0.91 ± 0.11	0.25	4.9
NH_3_	2	7.1 ± 2.8	0.31 ± 0.08	0.29 ± 0.03	0.08	1.6

*1 Methanol concentration conversion factor: 1 mM methanol = 48 mg-COD L^−1^ methanol.

*2 Values attained after 6 h of reactor operation.

### 3.2 Comparison of NH_2_OH and NH_3_ as electron donors for bioaugmented methanol production

Operation of the methanol production CSTR at 2 h HRT using NH_3_ as the electron donor resulted in a maximum methanol concentration of 7.1 ± 2.8 mg-COD_CH3OH_ L^−1^ (Fig. 2, bottom right panel). The maximum biomass specific CH_3_OH production rate achieved using NH_3_ (r_CH3OH−NH3_), 0.31 ± 0.08 mg-COD_CH3OH_ mg-COD_AOB_^−1^ d^−1^), was 24% of the corresponding maximum r_CH3OH−NH2OH_. This maximum r_CH3OH−NH3_ in the test CSTR is comparable with previous batch studies using mixed nitrifying populations (Taher and Chandran, 2013) or axenic *N. europaea* cultures (Hyman and Wood, 1983). The steady state r_CH3OH−NH3_ was slightly lower at 0.29 ± 0.03 mg-COD_CH3OH_ mg-COD_AOB_^−1^ d^−1^, which was 32% of the steady state value of r_CH3OH−NH2OH_.

The lower steady state methanol concentration and production rate using NH_3_ as the electron donor could be related to two factors. The first is the competition for AMO between CH_4_ and NH_3_ (Suzuki et al., 1976, Hyman and Wood, 1983, Keener and Arp, 1993). Second, co-oxidation of CH_4_ to CH_3_OH doesn’t result in a replenishment of reducing equivalents as would normally occur during NH_2_OH oxidation by HAO. In contrast, the external addition of NH_2_OH would obviate both competitive inhibition of AMO and any potential limitation of reducing equivalents. Nonetheless, methanol production rates utilizing NH_3_ in our research are comparable to those previously reported for AOB-mediated methanol production in batch reactors (Hyman et al., 1988, Taher and Chandran, 2013). Additionally, despite the potential of AMO inhibition by methane and methanol present in the CSTR, 65% of the NH_3_-N fed to the CSTR was oxidized to NO_3_-N at steady state (Fig. 2). This observation also links the biomethanol production process to a broader prospect that concurrent nitrification and methanol production using AOB is feasible.

From a practical perspective, ammonia is a near ideal electron donor for biogenic methanol production also considering that it is readily available, at no cost, in a WRRF. More importantly, the application of ammonia does not add any exogenous nitrogen loads into a WRRF in contrast to the use of NH_2_OH.

The biomethanol production rates obtained in this study using mixed-culture nitrifying activated sludge (Table 1) were also comparable to other studies employing pure or mixed cultures of AOB (ranging from 0.09 to 0.82 mg-COD_CH3OH_ mg-COD_AOB_^−1^ d^−1^) (Hyman and Wood, 1983, Hyman et al., 1988, Wang et al., 2010, Taher and Chandran, 2013) or MOB (ranging from 0.04 to 2.17 mg-COD_CH3OH_ mg-COD_MOB_^−1^ d^−1^) (Mehta et al., 1991, Takeguchi et al., 1997, Lee et al., 2004, Kim et al., 2010, Duan et al., 2011, Han et al., 2013, Kim et al., 2016). Furthermore, although MOB can convert methane to methanol, this option often requires MDH inhibitors as well as formate (Mehta et al., 1991, Takeguchi et al., 1997, Kim et al., 2010) to provide reducing equivalents, which may not be practically applicable in a WRRF.

For the different electron donors and HRT values employed in this study, the conversion efficiencies were determined based on methane supplied and methane transferred to the aqueous phase. The results showed that maximum 0.25% of methane supplied or 4.9% of methane transferred to the aqueous phase were converted to methanol under current experimental conditions (Table 1). These results present room for improving the process efficiency for methane conversion by further optimizing biogas supply and transfer rates.

### 3.3 Practical considerations for the application of bioaugmented methanol production platform

**Fig. 4:**
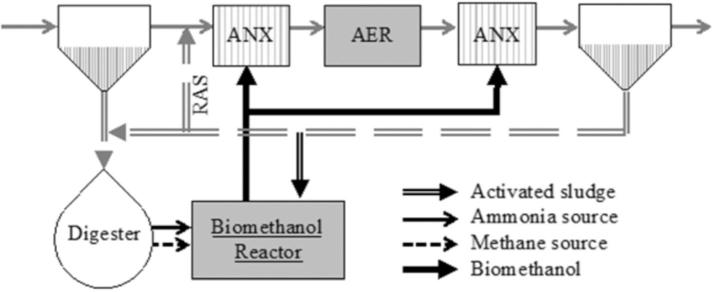
Schematic for potential integration of the bioaugmented methanol production platform into a WRRF. Biomethanol is produced in the sidestream nitrification process and fed to the pre- or post-anoxic zones in the mainstream process or used within the sidestream process itself. ANX: Anoxic zone. AER: Aerobic zone. RAS: Returned activated sludge.

In terms of integrating the AOB-mediated biomethanol production process within a WRRF, a range of ammonia-nitrogen and methane sources are available. Of these, sidestreams such as post-anaerobic digestion centrate and the anaerobic digester biogas stream itself are likely the most favorable to support this biogenic methanol production platform given the high substrate concentrations (ammonia and methane). Other *potentially available* methane sources within the WRRF could be the incoming wastewater, offgas from the sludge storage tanks or primary sludge thickeners (Daelman et al., 2012). However, apart from purchasing natural gas, the most easily *practically accessible* source of methane in a WRRF would be from anaerobic digestion. Over the longer term as WRRFs and landfills are co-located or WRRFs increasingly employ anaerobic processes for carbon recovery, some of the produced biogas could also be channeled towards biomethanol production. The biomethanol produced can be a beneficial supplemental carbon source used within the sidestream process itself, where it is produced or it can be channeled to the pre-or post-anoxic denitrification reactors in the mainstream process (Fig. 4).

Previous studies also have considered production of methanol for use as liquid fuel (Fei et al., 2014, Ge et al., 2014, Yang et al., 2014) or raw material for synthetic hydrocarbon products (Park and Lee, 2013, Fei et al., 2014). However, from a process integration perspective, the use of biomethanol produced in a WRRF for downstream denitrification is a likely optimal approach. A recent review also discusses the potential benefits to integrate AOB-catalyzed biomethanol process into existing WRRFs, in which the cost of methanol for denitrification can be a significant driver for biomethanol production process to be implemented (Lebrero and Chandran, 2018). Assuming 4.8 mg-COD_CH3OH_ mg-N_NO3_^−1^ is required for denitrification (Cherchi et al., 2009) and calculating a nitrogen normalized methanol production ratio (at 2 h HRT) using NH_3_ was 0.9 mg-COD_CH3OH_ mg-N_NH3_^−1^, the process described here can potentially offset ∼19% of the methanol requirement for denitrification.

While the supplementation of methane or produced methanol might raise the prospect of increasing methane- or methanol-utilizing bacterial concentrations in the activated sludge process, proper engineering controls could eliminate this possibility. For example, Yang et al. (2011) proposed that with a NH_4_^+^/CH_4_ molar ratio higher than 30, nitrogen would be non-limited for methanotrophs (nitrogen limitation occurs for NH_4_^+^/CH_4_ molar ratio <0.1) (Bodelier and Laanbroek, 2004). However, further increase in influent NH_4_^+^ concentrations would negatively impact methane oxidation by methanotrophs through competitive inhibition. Therefore, keeping a high influent NH_4_^+^/CH_4_ molar ratio should be considered to prevent the growth of methane oxidizing bacteria in the AOB-mediated biomethanol production process. On the other hand, it might be beneficial to promote the growth of methylotrophic denitrifying bacteria in the system to couple methanol production with methanol-supported nitrogen removal.

When applying the proposed process with actual biogas, AOB would also be impacted by constituents other than methane. Of these compounds, the inhibitory impact of hydrogen sulfide on AOB activity or nitrification in general could be a potential concern (Joye and Hollibaugh, 1995, Aesoy et al., 1998). While recent studies have evaluated the impacts of sulfide on mixed culture nitrifying biomass, most of the studies were aimed to achieve partial nitrification by inhibiting NOB with sulfide (Erguder et al., 2008, Beristain-Cardoso et al., 2010, Kouba et al., 2017, Seuntjens et al., 2018). Some studies have demonstrated that AOB were partially inhibited and were more resilient to sulfide inhibition than NOB (Bejarano Ortiz et al., 2013, Kouba et al., 2017). However, the specific impact of biogas constituents on AOB mediated biomethanol production has not been evaluated and is as such warranted. Nevertheless, even though we expect that AOB would be partially inhibited due to H_2_S present in the biogas, the methanol production platform would still be beneficial for the WRRFs considering the resilience of AOB to sulfide as demonstrated in Bejarano Ortiz et al., 2013, Kouba et al., 2017. Technology advances for the removal of hydrogen sulfide during biogas production (Krayzelova et al., 2015) can also help with the elimination of its negative impacts on methanol production. On the other hand, the presence of CO_2_ in biogas is expected to stimulate AOB metabolism (Jiang et al., 2015, Ma et al., 2015).

As examples of the economic benefits of this technology, the Blue Plains Wastewater Treatment Plant in Washington, DC spends approximately 5 million dollars per year for methanol (Sapno et al., 2003) and New York City spends $15-$20 million annually for external COD sources such as glycerol (Lebrero and Chandran, 2018). Were AOB-mediated methanol production to be considered at these plants, then based on our experimental results, the annual cost savings in terms of the internally produced methanol would be $0.15–0.2 million for Blue Plains and $ 0.45–0.8 million = for New York City. With further optimization, the savings could be improved. Moreover, it is important that the effluent ammonia-N concentration from the bioaugmented methanol production process be low enough to ensure robust operation of the liquid stream process and to meet the effluent limits. The process design in this study did not include denitrification, since the main focus was still AOB-mediated biomethanol production in a CSTR.

Since oxygen is required for this process, mixing methane and oxygen (flammable range 5–15%) might raise safety concerns. It is likely that successful implementation of the developed biomethanol production process will necessitate covered bioreactors with real-time monitoring and control of headspace gas composition in addition to similar monitoring of the pertinent aqueous chemical concentrations.

## 4 Conclusions

Results from this study demonstrate that bioaugmented methanol production from methane in a continuous flow process is both feasible and practicable using ammonia rather than hydroxylamine as the electron donor. This approach can potentially reduce the dependence on external carbon sources for denitrification as well as increase the utility of digester gas at WRRFs. Conversion of digester gas into methanol rather than flaring can also reduce the overall carbon footprints of the WRRFs.
