# Supplementary material for: Bioaugmented methanol production using ammonia oxidizing bacteria in a continuous flow process
Source: Bioresour Technol. 2019 May;279:101–7. doi: 10.1016/j.biortech.2019.01.092 (PMC6395879; doi:10.1016/j.biortech.2019.01.092)
Supplement: Supplementary data 1 [file mmc1.docx]

Supplementary Information:

Bioaugmented methanol Production using Ammonia Oxidizing Bacteria in a Continuous Flow Process

Yu-Chen Su^1,3^, Sandeep Sathyamoorthy^1,2,3^, Kartik Chandran^1,*^

^1^Columbia University

Department of Earth and Environmental Engineering

500 West 120^th^ Street

Room 1045 Mudd Hall

New York, NY 10027

^2^Current Address: Black & Veatch

2999 Oak Road, Suite 490

Walnut Creek, CA 94597

^3^co-first authors; contributed equally to this study

*corresponding author: kc2288@columbia.edu

**Performance of the nitrifying enrichment culture**

Concentrations of ammonia, nitrite, nitrate, and biomass were continuously monitored to assess the nitrification performance of the nitrifying enrichment culture. Initially the parent reactor was fed with 500 mg NH_3_-N L^-1^ and operated at 1-day HRT and 5-day SRT. To obtain higher AOB biomass concentration, the feed ammonia concentration was increased to 1000 mg NH_3_-N L^-1^ and the SRT was increased to 20-day. The parent reactor was disturbed by the changes of operation conditions and stabilized again in about 100 days. Biomass for experiment using NH_2_OH as electron donor and 7.5 h HRT (ED = NH_2_OH, HRT = 7.5 h) was collected during the transition period. For 2 h HRT experiments (ED = NH_2_OH or NH_3_, HRT = 2 h), biomass was collected after parent reactor nitrification performance was stabilized (Figure S1).

**Determination of bacterial concentrations based on total COD and qPCR**

Bacterial concentrations were calculated using reactor total COD concentration (tCOD) and AOB/NOB/heterotrophs fractions, which are determined based on qPCR results. Reactor total COD measured by COD digester vials are composed of AOB, NOB, non-nitrifying bacteria and inert concentrations. The active fraction of the nitrifying enrichment biomass with 20-d SRT was calculated assuming a specific decay rate (b) of 0.17 d^-1^ (Melcer et al., 2004) and a biomass debris fraction (f_d_) of 0.1 mgCOD/mgCOD (Grady et al., 1999).

$$Active fraction= \frac{1}{1+f_{d} \times b \times\theta_{c}}$$

Bacterial fractions are calculated based on the information that each AOB, NOB, and eubacteria cell contain 2.5 copies of *amoA* (Norton et al., 2001), 1 copy of NOB16S (Graham et al., 2007; Starkenburg et al., 2006), and 4.2 copies of EUB16S (Ferris et al., 1996), respectively, and mass of bacterial cells (Ahn et al., 2008; Farges et al., 2012).

Active bacterial concentration = Total COD x active fraction

AOB concentration = Active bacterial concentration x AOB fraction

**Optimization of feed air and methane flow rates**

The methane flow rate at 0.1 L/min was determined based on preliminary optimization of extant DO concentrations in the methane production process and the corresponding ultimate methanol concentrations achieved, as detailed below. Initially, the design of air and CH_4_ feed rates was aimed to maintain non-limiting DO concentrations and CH_4_ for both NH3 and CH4 oxidation to occur. Two conditions were tested during preliminary runs: 0.3 L/min air + 0.1 L/min CH_4_ and 0.1 L/min oxygen + 0.1 L/min CH_4_. The methanol production profiles for these two experiments were presented in supplementary information (Figure S2). The results showed that (1) high DO concentrations (at 10 mg O2/L) did not improve methanol production and (2) air flow rate can be lower (to increase the portion of aqueous methane). The authors acknowledge that there could be numerous more combinations of air and CH_4_ flow rates possible. However, given the trends observed with these flow rates tested, we selected 0.1L/min air and 0.1L/min CH_4_ for further experiments.

**Determination of the methane gas transfer coefficient**

The profile of the dissolved oxygen versus time was used to determine the gas transfer coefficient (k_L_a). Equation 1 was first used to describe the system, where represents the concentration of oxygen in the reactor and represents the saturation concentration of oxygen in water, given by the final oxygen concentration reached in the experiment.

| $\frac{dC_{r}}{\mathrm{dt}}=K_{L}a\times(C^{*}-C_{r)}$ |  | [1] |
| --- | --- | --- |

This equation was integrated to give

| $C_{r}=C^{*}\times e^{-K_{L}a\times t}$ | . | [2] |
| --- | --- | --- |

The k_L_a was calculated in Excel where the Solver function was used to minimize the squared error between the predicted concentration (using Equation 2 and the k_L_a) and the actual concentration obtained from the reactor. The oxygen K_L_a was further converted to methane K_L_a based on diffusivity and the relationship between K_L_a of oxygen and methane can be derived as follows:

$K_{L}a_{O2}=1.169\times K_{L}a_{CH4}$ [3](Yu et al., 2006)

K_L_a experiment apparatus was setup based on the same reactor configuration as the methanol production experiments i.e. 1.5L reactor with 0.2 L/h liquid feed. The reactor was first purged with nitrogen to evict dissolved oxygen, then 0.1 L/min air and 0.1 L/min nitrogen was supplied. Dissolved oxygen profiles were automatically recorded and used for K_L_a calculation (Figure S3).

(B)

(A)

**Figure S1.** Ammonia, nitrite, and nitrate concentrations (A) and biomass tCOD and calculated solids retention time (SRT) (B) of the nitrifying enrichment culture. The disturbance from 0 to 100 days was resulted from changes in operation conditions. The arrows indicated the period when biomass was collected for methanol production experiments. Error bars represent one standard deviation of duplicate measurements.

Figure S2. Methanol concentrations (A and B) and representative dissolved oxygen profiles (C and D) for methanol production experiments supply with different air flow rates or pure oxygen. Error bars indicate standard deviation of two replicate experiments. The methanol concentration profiles for Figure S2D are shown in Figure 2 in the main text.

Figure S3. Dissolved oxygen profiles obtained using equal volumetric flow rates of air and nitrogen (0.1 L/min each).

Table S1. Summary of studies evaluating biogenic methanol production using ammonia oxidizing bacteria.

| Description | Reactor  Type | Electron  Donor | Max S_MeOH_ ^*2^  mg-COD_CH3OH_ L^-1^ | Biomass Normalized MeOH Prod. Rate | Note | Ref. |
| --- | --- | --- | --- | --- | --- | --- |
|  |  |  |  | mg-COD_CH3OH_ mg-COD_AOB_^-1^ d^-1^ |  |  |
| *N. europaea* cell  suspension ^*1^ | Batch | NH_3_ | 27.3 | 0.80 | 1 mM NH_3_-N | (Hyman & Wood, 1983) |
|  |  | NH_3_ | 28.7 | 0.52 | 10 mM NH_3_-N |  |
| *N. europaea* cell  suspension ^*1^ | Batch | None | N/A | 0.10 |  | (Hyman et al., 1988) |
|  |  | NH_3_ | N/A | 0.59 | 10 mM NH3-N |  |
| *N. europaea* cell suspension ^*1,3^ | Batch | None | 12.4 | 0.09 |  | (Wang et al., 2010) |
|  |  | NH_3_ | 21.2 | 0.10 | 0.1 mM NH_3_-N |  |
|  |  | NH_3_ | 20.4 | 0.09 | 0.2 mM NH_3_-N |  |
| Nitrifying Enrichment Culture | Fed-batch | NH_3_ | 23.5 ± 0.5 | 0.21 | NH_3_ feed only | (Taher & Chandran, 2013) |
|  |  | NH_2_OH | 27.5 ± 0.8 | 0.30 | NH_2_OH feed only |  |
|  |  | NH_3_ +  NH_2_OH | 31.5 ± 1.2 | 0.22 | Simultaneous NH_3_ and NH_2_OH feed |  |
|  |  | NH_3_+  NH_2_OH | 40.7 ± 0.2 | 0.20 | Alternate NH_3_ and  NH_2_OH feed |  |
|  |  | NH_2_OH | 59.9 ± 1.1 | 0.82 | NH_2_OH feed only, with biomass replenishment |  |
| ^*1^ Biomass concentration conversion factors: 1 mg dry wt =1.42 mg-COD; 1 mg wet wt/mL = 344 mg-COD L^-1^.  ^*2^ Methanol concentration conversion factor: 1 mM methanol = 48 mg-COD L^-1^ methanol.  ^*3^ Also tested NH_2_OH-N as electron donor but NH_3_-N was found more effective. | | | | | | |

Table S2. Summary of studies evaluating biogenic methanol production using mixed methanotrophic cultures or axenic cultures of methane oxidizing bacteria (adapted from (Ge et al., 2014) and (Lebrero and Chandran, 2018)

| Bacterial strain | Process type | MDH inhibitor | Formate addition | Specific prod. rate | Ref |
| --- | --- | --- | --- | --- | --- |
|  |  |  |  | mg-COD_CH3OH_ mg-COD_MOB_^-1^ d^-1^ |  |
| *Methylosinus trichosporium* | Continuous (Immobilized cell) | MgCl2, phosphate | Yes | 2.17 | (Mehta et al., 1991) |
| *Methylosinus trichosporium* | Batch | cyclopropanol, phosphate | Yes | 1.21 | (Takeguchi et al., 1997) |
| *Methylosinus trichosporium* | Batch | NaCl | Yes | 0.28 | (Lee et al., 2004) |
| *Methylosinus trichosporium* | Batch | NaCl, EDTA | Yes | 1.16 | (Kim et al., 2010) |
| *Methylosinus trichosporium* | Batch (membrane aerated) | MgCl2, phosphate | Yes | 0.04 | (Duan et al., 2011) |
| Methantrophic consortium from landfill site | Continuous | NaCl | No | 3.52 | (Han et al., 2013) |
| *Methylocystis bryophila* | Batch | MgCl2, phosphate | Yes | 0.07 | (Kim et al., 2016) |

**REFERENCES**

1. Ahn, J.H., Yu, R., Chandran, K., 2008. Distinctive microbial ecology and biokinetics of autotrophic ammonia and nitrite oxidation in a partial nitrification bioreactor. Biotechnol. Bioeng 100,1078-1087
2. Duan, C., Luo, M., Xing, X., 2011. High-rate conversion of methane to methanol by Methylosinus trichosporium OB3b. Bioresour. Technol. 102,7349-7353
3. Farges, B., Poughon, L., Roriz, D., Creuly, C., Dussap, C.G., Lasseur, C., 2012. Axenic Cultures of Nitrosomonas europaea and Nitrobacter winogradskyi in Autotrophic Conditions: a New Protocol for Kinetic Studies. Appl. Biochem. Biotechnol. 167, 1076-1091
4. Ferris, M.J., Muyzer, G., Ward, D.M., 1996. Denaturing gradient gel electrophoresis profiles of 16S rRNA-defined populations inhabiting a hot spring microbial mat community. Appl. Environ. Microbiol. 62, 340-346
5. Ge, X., Yang, L., Sheets, J.P., Yu, Z., Li, Y., 2014. Biological conversion of methane to liquid fuels: status and opportunities. Biotechnol. Adv. 32, 1460-1475
6. Grady, C.P.L.J., Daigger, G.T., Lim, H.C. 1999. Biological Wastewater Treatment. New York: Marcel Dekker
7. Graham, D.W., Knapp, C.W., Van Vleck, E.S., Bloor, K., Lane, T.B., Graham, C.E., 2007. Experimental demonstration of chaotic instability in biological nitrification. ISME J. 1, 385-93
8. Han, J.-S., Ahn, C.-M., Mahanty, B., Kim, C.-G., 2013. Partial Oxidative Conversion of Methane to Methanol Through Selective Inhibition of Methanol Dehydrogenase in Methanotrophic Consortium from Landfill Cover Soil. Appl. Biochem. Biotechnol. 171, 1487-1499
9. Hyman, M.R., Wood, P.M., 1983. Methane oxidation by Nitrosomonas europaea. The Biochem. J 212, 31-37
10. Hyman, M.R., Murton, I.B., Arp, D.J., 1988. Interaction of ammonia monooxygenase from Nitrosomas europaea with alkanes, alkenes, and alkynes. Appl. Environ. Microbiol. 54, 3187
11. Kim, H.G., Han, G.H., Kim, S.W., 2010. Optimization of lab scale methanol production by Methylosinus trichosporium OB3b. Biotechnol. Bioprocess Eng. 15, 476-480
12. Kim, I.-W., Lee, J.-K., Kim, S.-Y., Mardina, P., Patel, S.K.S., 2016. Biological Methanol Production by a Type II Methanotroph Methylocystis bryophila. J. Microbiol. Biotechnol. 26, 717-724
13. Lebrero, R., Chandran, K., 2018. Biological conversion and revalorization of waste methane streams. Crit. Rev. Env. Sci. Tec 47, 2133-57
14. Lee, S.G., Goo, J.H., Kim, H.G., Oh, J.-I., Kim, Y.M., Kim, S.W., 2004. Optimization of methanol biosynthesis from methane using Methylosinus trichosporium OB3b. Biotechnol Lett. 26, 947-50
15. Mehta, P.K., Ghose, T.K., Mishra, S., 1991. Methanol biosynthesis by covalently immobilized cells of *Methylosinus trichosporium*: Batch and continuous studies. Biotechnol. Bioeng. 37, 551-556
16. Melcer, H., Dold, P.O., Jones, R.M., Bye, C.M., Stensel, H.D., et al. 2004. Methods for Wastewater Characterization in Activated Sludge Modelling, Water Environment and Reuse Foundation, Alexandria, VA
17. Norton, J.M., Alzerreca, J.J., Suwa, Y., Klotz, M.G., 2001. Diversity of ammonia monooxygenase operon in autotrophic ammonia-oxidizing bacteria. Arch. Microbiol. 177, 139-149
18. Starkenburg, S.R., Chain, P.S.G., Sayavedra-Soto, L.A., Hauser, L., Land, M.L., et al. 2006. Genome Sequence of the Chemolithoautotrophic Nitrite-Oxidizing Bacterium Nitrobacter winogradskyi Nb-255. Appl. Environ. Microbiol. 72, 2050-2063
19. Taher, E., Chandran, K., 2013. High-Rate, High-Yield Production of Methanol by Ammonia-Oxidizing Bacteria. Environ. Sci. Technol 47, 3167-3173
20. Takeguchi, M., Furuto, T., Sugimori, D., Okura, I., 1997. Optimization of methanol biosynthesis by *Methylosinus trichosporium* OB3b: An approach to improve methanol accumulation. Appl. Biochem. Biotech. 68, 143-152
21. Wang, L., Tabata, K., Kamachi, T., Okura, I., 2010. Effect of Electron Donor on Methanol Production by Ammonia-oxidizing Bacterium *Nitrosomonas europaea*. J. Jpn. Petrol. Inst. 53, 319-326
22. Yu, Y., Ramsay, J.A., Ramsay, B.A. 2006. On-Line Estimation of Dissolved Methane Concentration During Methanotrophic Fermentations. Biotechnol. Bioeng. 95, 788-793
